# Commentary: Case report: Paraganglioma masquerading as angiosarcoma: diagnostic‐dilemma in vascular tumors

**DOI:** 10.3389/fonc.2026.1669341

**Published:** 2026-03-12

**Authors:** Tomonori Kawasaki, Kojiro Onohara, Yosuke Horita, Masataka Hirasaki, Satoshi Kanno, Masanori Wako, Jiro Ichikawa

**Affiliations:** 1 Department of Pathology, Saitama Medical University International Medical Center, Hidaka, Japan; 2 Department of Diagnostic Radiology, Interdisciplinary Graduate School of Medicine, University of Yamanashi, Chuo, Japan; 3 Department of Medical Oncology, Saitama Medical University International Medical Center, Hidaka, Japan; 4 Department of Clinical Cancer Genomics, Saitama Medical University International Medical Center, Hidaka, Japan; 5 Department of Orthopedic Surgery, Interdisciplinary Graduate School of Medicine, University of Yamanashi, Chuo, Japan

**Keywords:** paraganglioma, angiosarcoma, imaging, histopathological findings, retroperitoneal

## 1 Introduction

Paragangliomas are a unique subset of neuroendocrine neoplasms (NEN) with distinct functional and genetic characteristics (1). We were greatly intrigued by the recent publication by Rajoo et al., titled “Case report: Paraganglioma masquerading as angiosarcoma: diagnostic dilemma in vascular tumors,” (2) and we deeply appreciate the authors for reporting this valuable case. In this study, we aimed to discuss the pathological differences that made distinguishing this case from angiosarcoma challenging, as well as to describe the CT findings of paraganglioma and its differentiation.

### 1.1 Subsections relevant for the subject

1. Consideration of Paraganglioma and its differential diagnoses based on CT findings.
2. Consideration of Paraganglioma and its differential diagnoses based on pathological findings.

## 2 Commentary and discussion

### 2.1 Imaging

Paragangliomas and Pheochromocytoma are tumors that can produce norepinephrine and dopamine, which may cause various symptoms such as headaches, palpitations, excessive sweating, anxiety, tremors, and nausea, enabling differential diagnosis in such cases (3). However, they can also be asymptomatic and incidentally discovered during imaging, as noted in this study. Common imaging techniques include CT and MRI, while single-photon emission computed tomography and [18F]-FDOPA positron emission tomography are valuable for staging assessment (3). Regarding the CT findings in this case, it is recommended that the anatomical labels indicated by the arrows be corrected because they are inconsistent. Specifically, in Figure 1A, the arrow points to the aorta, not the tumor; in Figure 1B, the tumor is actually the right kidney; in Figure 1C, the labels should be revised to show that the aorta is the IVC, the IVC is the tumor, and the tumor is the aorta. Based on the contrast effects in normal organs, Figures 1A, B likely correspond to the arterial phase, whereas Figure 1C corresponds to the equilibrium phase.

The contrast pattern of CT in paragangliomas has been reported as “fast-in-fast-out,” characterized by obvious enhancement during the arterial and venous phases, followed by a gradual decrease in the delayed phases (4). In this case, although the exact CT values were not available, highly vascular features comparable to those of the aorta or renal cortex were not observed during the arterial phase, differing slightly from previous reports (4). However, the evaluation of early contrast enhancement is insufficient (the possibility that the washout process has already occurred cannot be excluded). Nevertheless, the lack of developed vascular structures around or inside the lesion may have made it difficult to consider paraganglioma in the differential diagnosis (5). On the contrary, as mentioned in the Discussion, the heterogeneous contrast enhancement suggests the possibility of internal degeneration, hemorrhage, or necrosis. Evaluating and presenting these pathological findings will greatly ensure the accuracy of the authors’ discussions. Furthermore, considering that the area around the abdominal aorta is a relatively common site for paragangliomas and the possibility of internal degeneration, hemorrhage, or necrosis in hypervascular tumors, paragangliomas should be included in the differential diagnosis (5). For the angiosarcomas considered in this study, enhanced CT features included uneven central enhancement, with marginal enhancement during the arterial phase. The enhancement area extends to the center of the lesion during the delayed phase and becomes more pronounced than during the arterial phase (6). In Rajoo’s case, we also considered schwannoma in the differential diagnosis based on the contrast CT findings. The comparison of imaging characteristics depends on individual cases. On non-contrast CT, paragangliomas showed an average CT value of 41, whereas schwannomas showed an average CT value of 35. Schwannomas typically display weak enhancement during the arterial phase but demonstrate a slow-persistent pattern, where enhancement gradually increases from the venous phase to the delayed phase (4).

### 2.2 Pathological findings in angiosarcoma and paragangliomas

Notably, the pathological findings of the biopsy sample suggested an angiosarcoma. AS is a highly aggressive malignant vascular tumor that accounts for approximately 2–4% of all sarcomas. More than 50% originate subcutaneously but can also arise in deep tissues, breasts, bones, and other sites (7). Secondary AS is associated with radiotherapy and long-term lymphedema (7). Histopathologically, they range from well-differentiated cases resembling hemangiomas to poorly differentiated forms lacking evident vascular structures composed of solid sheets of spindled, epithelioid, round, or anaplastic cells (8). The useful IHC markers include a combination of CD31, CD34, D2-40, VE-cadherin, and VEGFR. In terms of staining patterns, CD31, D2-40, and VEGFR show positivity in both the membrane and cytoplasm; CD34 is positive in the membrane, while VE-cadherin is positive in the cytoplasm (8). Strong nuclear staining with ERG or FLI1 is also useful (8). The pathological differential diagnosis varies based on the location and subtype of AS. For example, in epithelioid AS, differential diagnoses include epithelioid hemangioma, epithelioid hemangioendothelioma, carcinomas, melanomas, and epithelioid malignant mesenchymal tumors, such as sclerosing epithelioid fibrosarcoma and epithelioid sarcoma (8). As paraganglioma and AS are typically not differentiated, presenting biopsy samples and clarifying how H&E and IHC findings are used to differentiate AS holds significant value and is likely to contribute to the understanding of the pathology of AS and paraganglioma, benefiting the elucidation of the essence of these two diseases. The Zellballen pattern is the most commonly identified paraganglioma cellular structure. Variants include angiomatous and sclerosing types as well as small-cell variants resembling other carcinomas. The IHC patterns were negative for keratin AE1/AE3, keratin 8 (CAM5.2), and TTF1, but positive for GATA3, tyrosine hydroxylase, synaptophysin, SOX10, and S100 (1, 9). However, these expression patterns vary depending on the location of the paraganglioma and require careful interpretation (9). The pathological differential diagnoses include metastatic carcinoma, neuroendocrine tumors, and melanoma (10). While paragangliomas are recognized as malignant tumors, they often follow a course similar to that of benign tumors because of their low metastasis rates (1). Among these, retroperitoneal paragangliomas are considered to have higher malignant potential, necessitating long-term follow-up (10). In retroperitoneal PGL, features such as endovascular tumor embolus, S100 negativity, and succinodehydrogenase subunit B negativity have been reported to correlate with progression-free survival (11). Predicting malignancy is challenging, because no single histological finding or biomarker exists. Thus, scoring systems based on morphological features, the Ki67 proliferation labeling index, and tumor biochemical profiles have been proposed (3).

## 3 Conclusions

In this study, we examined the imaging findings and differential diagnoses of paraganglioma and AS. Although paragangliomas are most commonly found in the retroperitoneal region, an accurate diagnosis requires histological examination. Because the two diseases considered for differential diagnosis in this case were not typically distinguishable, presenting biopsy samples to demonstrate the pathological evaluations that led to the suspicion of AS would enhance the value of this study.

## References

[1] Mete O Juhlin CC . Recent progress in the pathologic classification of pheochromocytomas and paragangliomas. Best Pract Res Clin Endocrinol Metab. (2024) 38:101958. doi: 10.1016/j.beem.2024.101958, PMID: 39609157

[2] Rajoo AR Kannairan S Habeebullah Khan HA Idris MA Tan GC Chandra Sakaran KR . Case report: Paraganglioma masquerading as angiosarcoma: Diagnostic-dilemma in vascular tumors. Front Oncol. (2024) 14:1462956. doi: 10.3389/fonc.2024.1462956, PMID: 39703846 PMC11655487

[3] Brewczyński A Kolasińska-Ćwikła A Jabłońska B Wyrwicz L . Pheochromocytomas and paragangliomas-current management. Cancers (Basel). (2025) 17:1029. doi: 10.3390/cancers17061029, PMID: 40149362 PMC11941679

[4] Cao Y Wang Z Ren J Liu W Da H Yang X . Differentiation of retroperitoneal paragangliomas and schwannomas based on computed tomography radiomics. Sci Rep. (2023) 13:9253. doi: 10.1038/s41598-023-28297-6, PMID: 37286581 PMC10247726

[5] Ji XK Zheng XW Wu XL Yu ZP Shan YF Zhang QY . Diagnosis and surgical treatment of retroperitoneal paraganglioma: A single-institution experience of 34 cases. Oncol Lett. (2017) 14:2268–80. doi: 10.3892/ol.2017.6468, PMID: 28789448 PMC5530091

[6] Yu JF Cui H Ji GM Li SQ Huang Y Wang RN . Clinical and imaging manifestations of primary cardiac angiosarcoma. BMC Med Imaging. (2019) 19:16. doi: 10.1186/s12880-019-0318-4, PMID: 30764784 PMC6375141

[7] Thway K Billings SD . Angiosarcoma. Who Classification of Tumours. 5th ed. Lyon: IARC Press (2020) p. 176–8.

[8] MaChado I Giner F Lavernia J Cruz J Traves V Requena C . Angiosarcomas: Histology, immunohistochemistry and molecular insights with implications for differential diagnosis. Histol Histopathol. (2021) 36:3–18. doi: 10.14670/HH-18-246, PMID: 32885407

[9] Mamilla D Manukyan I Fetsch PA Pacak K Miettinen M . Immunohistochemical distinction of paragangliomas from epithelial neuroendocrine tumors-gangliocytic duodenal and cauda equina paragangliomas align with epithelial neuroendocrine tumors. Hum Pathol. (2020) 103:72–82. doi: 10.1016/j.humpath.2020.07.010, PMID: 32668278 PMC7530041

[10] Ntanasis-Stathopoulos I Tsilimigras DI Klapsinou E Daskalopoulou D Vaida S Arnogiannaki N . Challenging differential diagnosis of an extra-adrenal paraganglioma; the role of fine needle aspiration cytology. Diagn Cytopathol. (2017) 45:565–8. doi: 10.1002/dc.23696, PMID: 28261927

[11] Chun C Song L Xu G Shi Q Li F Jia X . Analysis of clinical and pathological characteristics of retroperitoneal paraganglioma and associated prognostic factors. J Surg Oncol. (2024) 130:47–55. doi: 10.1002/jso.27681, PMID: 38864273

